# Organosolv Lignin-Based Wood Adhesive. Influence of the Lignin Extraction Conditions on the Adhesive Performance

**DOI:** 10.3390/polym8090340

**Published:** 2016-09-14

**Authors:** Issam Dababi, Olinda Gimello, Elimame Elaloui, Françoise Quignard, Nicolas Brosse

**Affiliations:** 1 Laboratoire d’Etudes et de Recherche sur le Matériau Bois, Faculté des Sciences et Technologies, Université de Lorraine, EA 4370, Boulevard des Aiguillettes, BP 70239, 54506 Vandœuvre-Lès-Nancy CEDEX, France; dababiissam@yahoo.fr; 2 ICGM, UMR 5253 CNRS--ENSCM-UM, Matériaux Avancés pour la Catalyse et la Santé, 8 Rue de l’Ecole Normale, 34296 Montpellier CEDEX 5, France; olinda.gimello@enscm.fr (O.G.); francoise.quignard@enscm.fr (F.Q.); 3 Laboratoire Matériaux, Energie et Environnement UR14ES26, Faculté des Sciences de Gafsa, Université de Gafsa, 2100 Gafsa, Tunisie; limam_aloui@yahoo.fr

**Keywords:** wood adhesive, tannin, lignin, organosolv, Lewis acid

## Abstract

Ethanol organosolv alfa grass lignins were extracted in the presence of sulfuric acid or Lewis acids (Sc(OTf)_3_, FeCl_3_) as catalysts and subjected to a comprehensive structural characterization by solid state ^13^C NMR, GPC, MALDI-TOF, and ASAP-MS/MS. The impact of the severity of the treatment and of the nature of the acid catalyst on the recovered lignin structure was investigated. The lignins isolated at high severity were highly recondensed and partly composed of regular structures composed of furan-like rings. The alfa (*Stipa tenacissima* L.) organosolv lignins were used for the preparation of formaldehyde-free adhesives which were characterized by TMA and used for the preparation of particleboard without any addition of synthetic resin. It has been demonstrated for the first time that: (1) the addition of 10% to 30% of organosolv alfa lignin in a tannin-based adhesive improved the adhesive performance; and (2) the conditions of the lignin extraction strongly impact the lignin-based adhesive performances. The highly recondensed lignin extracted with sulfuric acid as a catalyst allowed the production of resins with improved performances. Formulations composed of 50% glyoxalated alfa lignin and 50% of Aleppo Pine tannins yielded good internal bond strength results for the panels (IB = 0.45 MPa) and satisfied relevant international standard specifications for interior-grade panels.

## 1. Introduction

The production of environmentally friendly wood adhesive has been a topic of interest since 1970s [1,2]. Tannins-based formaldehyde-free adhesives have been successfully described and used for interior and exterior wood bonding. However, the production of tannin-based adhesives is limited by the relatively limited supply of tannins. Thus, the industrial development of such green wood adhesives depends on the availability of low-price, high quality, and reactive biopolymers.

Lignin is one of the most abundant natural and renewable polymers and is currently a co-product of pulp industries. The occurrence of large volumes of low value lignin has made it a potentially attractive material for adhesive production. Utilization of lignin as a partial substitute or extender for phenolic wood adhesives has been extensively described [3,4,5,6]. Nevertheless, in contrast with the great number of academic publications and patents regarding lignin utilizations, the industrial uses of lignin for material applications are scarce. Thus, due to the low purity and quality and to the large variability of lignin, industrial utilization of lignin in the adhesive field is still an unsolved problem.

Beside the pulp and paper sector, lignin is expected to be produced in the near future as a co-product of the cellulosic biofuel industry [7]. To improve the cost-effectiveness of bioethanol production, a widely studied strategy is the biorefinery concept in which all components, including lignin, are used in different sectors of activities. Among all the pretreatments studied for bioethanol production, the organosolv process seems promising since it produces not only a cellulosic pulp displaying a good enzymatic digestibility but also sulfur-free lignin fractions potentially usable for material applications [8,9]. Sulfuric acid is the most commonly employed catalyst for the organosolv pretreatment, but recently Lewis acids have been also studied [10].

Characterization of lignin structure is an important and difficult issue. It is widely accepted that lignin is a complex 3-D crosslinked polymer which has a high degree of variability in its structure. Beside NMR techniques which have been shown to be reliable and robust methods, mass spectrometry using soft MS ionization techniques have been described to unravel the complex architecture of lignin macromolecules [11]. The structural characterization of organosolv wheat straw lignin has been previously published using atmospheric pressure chemical ionization mass spectroscopy and tandem mass spectroscopy (MS/MS). Unlike the generally described random structure, the authors described a linear lignin polymer composed of repetitive units with a molecular mass in the range of 600–1800 Da [12,13].

Previous works reported the production of tannin/organosolv lignin wood resins. Because of the low reactivity of lignin toward cross linking reactions, a pre-reaction with glyoxal has been proposed [3,4,5]. In a previous paper, we described a lignin based adhesive composed of 40% of miscanthus organosolv lignin and of 99.5% of renewable materials [6]. However, the ability of lignin polymer to make significant impact as substitute of tannins in green adhesives depends on a better control of the molecular structure and variability of the lignin which is a function of the nature of the raw material but also of the processes used for the lignin extraction. We previously studied the effect of the severity of an ethanol organosolv pretreatement (sulfuric acid conc., temperature, duration) on the chemical structure of the extracted lignin. As a result, the severity of the treatment largely impacts the molecular mass and the phenolic and aliphatic OH contents of the recovered material [8,9]. In the same way, it has been recently shown that the nature of the acid catalyst employed in an organosolv treatment influenced the chemical characteristic of the organosolv lignin. Utilization of Lewis acids in an organosolv pulping has been successfully described starting from wheat straw. It has been shown the nature and the hardness of the Lewis acid also influenced the structure of the recovered lignins [10].

Alfa grass (*Stipa tenacissima* L.), also called Esparto grass is a perennial plant of northern Africa. It is the main raw cellulosic material for the pulp industry in Algeria and Tunisia, using the soda process [14]. Soda lignin isolated from alfa grass has been already characterized and used as filler in a phenolic composite. Acetosolv alfa lignin has also been characterized [15,16], but to the best of our knowledge, ethanol organosolv lignin has never been described starting from alfa grass.

In this paper, we described the ethanol organosolv extraction of lignin from alfa grass. Sulfuric acid and two different Lewis acids were evaluated as catalyst of the organosolv pulping. The impact of the severity of the treatment and of the catalyst used on the chemical structure of the recovered lignin was evaluated using ^13^C and ^31^P nuclear magnetic resonance (NMR), matrix-assisted laser desorption/ionization time-of-fight mass spectrometry (MALDI TOF), Atmospheric Solids Analysis Probe tandem mass spectrometry (ASAP-MS/MS), and gel permeation chromatography (GPC). The organosolv lignin was used as a component of 99.5% green adhesive formulation without formaldehyde, the formulation containing also sumac tannins. Aleppo Pine (*Pinus halepensis*), is a pine native to the Mediterranean region. Aleppo pine tannins/alfa lignin-based formaldehyde-free particleboards displaying good performance were prepared and tested.

## 2. Materials and Methods

### 2.1. Materials

Alfa grass (holocellulose 74% ± 3% hemicelluloses, lignin 23% (Klason lignin 20.2% ± 0.4%) and acid soluble lignin 2.8% ± 0.1%, total extractive 11.5% (ethanol-toluene 8% and hot water extractives 4.7%), proteins 1.25%, and ashes 1.3%) was collected from Gafsa (Tunisia) in October 2014, dried in a dark place at room temperature, and then cut into small pieces (1 cm). Aleppo Pine (*Pinus halepensis*) barks were purchased from a local Tunisian market. Metal salts and reagents were purchased from Sigma-Aldrich. All chemical used were of analytical or reagent grade (Sigma-Aldrich, Saint Louis, IN, USA). The determination of the Klason lignin was performed in duplicate following published procedure [17].

### 2.2. Lignin Extraction

Extraction of tannin from barks was based on published procedures [18,19]. Extraction was carried out in water containing 2% of sodium sulfite and 0.5% of sodium bicarbonate, with a sample:water ratio equal to 1:10 under continuous stirring for 6 h at 70 °C. The extract was air dried at 50 °C until constant weight. Lignin was extracted from alfa grass by an ethanol-organosolv prossess according to a published procedure [10]. The organosolv treatments were performed using a large range of combined severities (CS from 1.08 to 3.12) calculated using the equation below: *Combined severity* CS = log [*t* × exp ((*T* − 100)/14.7)] − pH; (*t* = time in mn, *T* = temperature in °C, the pH of the ethanol-water solutions were determined using a pH-meter) [20].

Two Lewis acids (FeCl_3_, Sc(OTf)_3_) (and H_2_SO_4_ in the same conditions for comparison) were also used as catalyst (4 mmol·L^−1^). A typical experiment is given: 40 g of alfa grass was mixed in 1.25 L aqueous ethanol (EtOH 65%, H_2_O 35%) in a 2 L autoclave (Autoclave France^®^, Rantigny, France). The mixture was stirred for 2 h at 160 °C. The reactor was cooled at room temperature. Pulp and black liquor were separated using a nylon filter. The pulp was washed three times with warm (60 °C) ethanol/water solution 65/35 (3 × 300 mL). Three volumes of water were added to precipitate the ethanol organosolv lignins (L1–L11), which were collected by centrifugation and air dried.

### 2.3. Lignin Characterization

#### 2.3.1. Size Exclusion Chromatography

The weight average molecular weights (*M*_w_) of lignin were measured using size exclusion chromatography (SEC) on a Shimadzu UFLC chromatograph (Kyoto, Japan) equipped with a RI refractive index detector and a PolarGel-M column (300 mm × 7.5 mm). The eluent was DMF (+LiCl 0.05 wt %) at 40 °C with a flow rate of 1 mL/min and the polystyrene standards were used for calibration. The lignin samples were dissolved at 10 mg/mL in DMF (+LiCl 0.05 wt %) and the solutions were filtered to 0.45 µm with a PTFE filter before analysis.

#### 2.3.2. Solid State ^13^C NMR and ^31^P NMR Spectroscopies

^13^C NMR solid state cross-polarization/magic-angle spinning spectroscopy (^13^C CPMAS-NMR) was performed on a Bruker AV-300 (Billerica, MA, USA) operating at 75 MHz. The spectra were recorded applying the following parameters: 4000 Hz rotor spin rate, 1 ms of contact time, 20 ms of acquisition time, 3000 scans. Deconvolution analysis was performed according to [21]. ^31^P NMR spectra were acquired after derivatizing 25 mg lignin with 2-chloro-4,4,5,5-tetramethyl-1-1,3,2-dioxaphospholane. Cyclohexanol was used as an internal standard. Quantitative NMR spectra were acquired using an inverse-gated decoupling pulse sequence with a 30° pulse angle and 25 s pulse delay [8].

#### 2.3.3. MALDI-TOF Mass Analysis

Full scan mass spectra MALDI-TOF-MS were performed on a MALDI-TOF/TOF Bruker Ultraflex III mass spectrometer (Billerica, MA, USA) using a nitrogen laser for MALDI (λ = 337 nm). Mass spectra of 2500 shots were accumulated for the spectra at a 25 kV acceleration voltage and reflectron lens potentials at 26.3 KV. Mixture of peptides was used for external calibration. The lignin samples were dissolved at 20 mg/mL in a mixture of acetonitrile:ethanol:water (50:40:10 vol %). The matrix used was DHB (2,5-dihydroxybenzoic acid) and it was dissolved at 10 mg/mL in a mixture of acetone and water (50:50 vol %). The cationization agent was LiCl (10 mg/mL in methanol) for lignin samples L10 and L11. For the lignin sample L9 the agent of cationization used was NaCl. 10 µL of matrix solution, 4 µL of lignin sample, and 1 µL of salt were mixed. 1 µL of this mixed solution was hand spotted on a MALDI target and left to dry before analysis. For all spectra recorded in positive ionization mode, the most abundant ion was the protonated adducts [M + H]^+^.

#### 2.3.4. Atmospheric Solids Analysis Probe Tandem Mass Spectrometry (ASAP-MS/MS)

ASAP-MS/MS analyses were performed on a SYNAPT G2 HDMS QTOF Mass Spectrometer fitted with an Atmospheric Solids Analysis Probe (Waters, Milford, CT, USA). The samples were deposited directly to the exterior of a glass capillary that is attached to the ASAP probe. The sample in the gas phase was ionized by the proximity to the discharge of a corona needle. Ions were then passed from the atmospheric pressure region to the mass spectrometer. A nitrogen gas flow of 500 L/h was ramped from 50 to 650 °C at 200 °C/min for thermal desorption. The corona discharge voltage was 4 µA and the sampling cone voltage was 40 V. For MS/MS experiments, the collision energy in the transfer cell was set to 35 eV. ASAP MS/MS mass spectra were acquired in positive ion mode for the [M + H]^+^ ions at *m*/*z* 331 and at *m*/*z* 509 and ASAP MS/MS mass spectra in negative ion mode for the [M − H]^−^ ions at *m*/*z* 329 and at *m*/*z* 507.

### 2.4. Resin Adhesive Preparation

To prepare glyoxalated lignin, the process described by El Mansouri et al. [3] was used with slight modifications. 1.6 g of alfa lignin was gradually added to 2.11 g of water. Sodium hydroxide solution (30%) was added periodically to maintain the pH of the solution between 12 and 12.5 for better dissolution of the lignin powder. The mixture was stirred and heated to 65 °C, after 30 min, 0.96 g of glyoxal (40% in water) was added with a magnetic stirrer on a hot plate for 8 h. A 45 wt % concentration of Aleppo pine tannin solution in water was prepared and pH adjusted to 10 with NaOH 33% water solution. Hexamine at 33% solution in water was added (6 wt/wt % solids) as a hardener. Then the glyoxalated lignin was mixed with this tannin solution. Five formulations for each lignin have been tested (glyoxalated lignin/tannin from 10% to 50%).

### 2.5. Thermomechanical Tests

All tests were performed by TMA in triplicate under the following conditions. The hardening reaction of the resin was evaluated by TMA by the study of the rigidity of the wood–resin joint as a function of temperature. The samples were prepared by applying 22 mg of resin system between two beech wood plies in a layer of 350 µm, for total sample dimensions of 21 × 6 × 1.1 mm^3^ (moisture content = 12%). These beech-resin-beech specimens were tested in non-isothermal mode between 40 and 220 °C (heating rate 10 °C/min). The thermomechanical analyzer was a TMA40 instrument from Mettler Toledo (Columbus, OH, USA). The software used for data treatment was STARe (Mettler Toledo, Columbus, OH, USA). Deflection curves, which permitted the determination of the modulus of elasticity (MOE), were obtained in a three-point flexion mode. The MOE of wood–resin systems give a good indication of the strength of the final application of the experimental glue. The maximum MOE value and its increase as a function of time or temperature for wood–resin systems give a good indication of the possible end performance of the adhesive system tested.

### 2.6. Particleboard Manufacture and Testing

One-layer laboratory particleboards of 350 × 300 × 16 mm^3^ dimension were prepared using only core particles of beech (*Fagus sylvatica*) and Norway spruce (*Picea abies*) wood particles (moisture content = 3%) at 0.36 KPa maximum pressure and 190–195 °C press temperature. The total adhesive resin solids load on dry wood was 10% *w*/*w* of the total mix of glyoxalated + lignin tannin. The total pressing time was 7.5 min. The particleboards were tested for dry internal bond (IB) strength test in triplicate, which is a relevant international standard test (EN 312).

## 3. Results

The lignin was extracted from alfa grass using ethanol organosolv processes (Table 1, L1–L8). The influence of two parameters (i.e., temperature and sulfuric acid concentration) was examined. Considering previous studies [22], an ethanol concentration of 65% and duration of the treatment of 1 h were selected. Two Lewis acids (Sc(OTf)_3_ and FeCl_3_) have also been studied as catalyst and compared with sulfuric acid. L9 to L11 were carried out using the comparable conditions (2 h, pH = 2.4) and the same combined severity (CS = 1.45). Table 1 presents the experimental conditions, the combined severity values, the lignin yields, and the molecular masses (*M*_w_).

As previously described by Goh et al. [22], it was observed from L1 to L8 that for the same duration of treatment an increase of the severity resulted in an increase in the lignin extraction from the pulp. Satisfactory lignin yields were obtained from L4 to L8 (>15%) with a lignin purity (Klason lignin) higher than 95% (data not shown). The molecular mass of the lignin fractions, estimated by Size Exclusion Chromatography, are in accordance with previous results [9]. From Table 1 (L1–L8), it can be seen that the molecular mass decreased with the severity: the higher weight (*M*_w_ > 10,000) were isolated at low severity (CS = 1–1.5) and at low temperature (*T* ≈ 160 °C). This observation confirms previous works showing that the organosolv pulping extensively cleaved aryl-ether linkages in lignin network and that this degradation is a function of the pulping conditions [8,9]. It also can be seen that, at a constant temperature of 185 °C (L3 to L5), an increase in sulfuric acid concentration from 0% to 1.7% resulted in an increase in the molecular weight of the lignin fragments. This observation can be rationalized by the well-known acid-catalyzed lignin recondensation primarily involving the aromatic C5 of coniferyl units [23].

From L9 to L11, it can be observed that for the three organosolv processes performed in comparable conditions, Lewis acids lead to lower lignin yields (≈10%) compared to sulfuric acid (≈13%). Moreover, smaller lignin molecular masses were observed for the L10 and L11. These results confirm that Lewis acids promote extensive β-O-4 cleavage. The lowest molecular mass obtained for Sc(OTf)_3_ is in accordance with a previous paper showing that using this acid, a lower recondensation of the lignin was observed, the harder the Lewis acid, the lower the lignin molecular mass [10].

**Figure 1:**
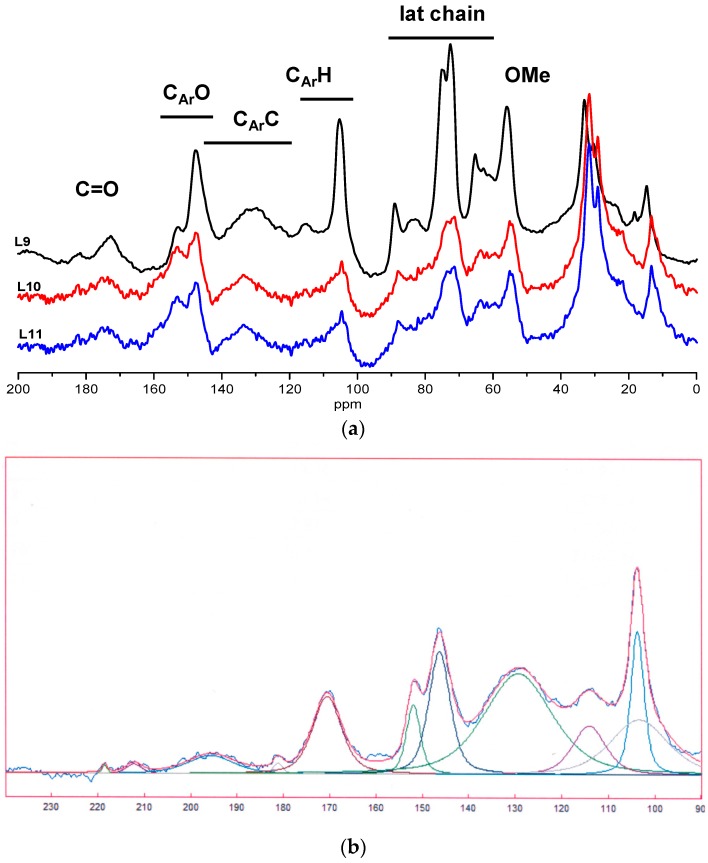
(**a**) Solid-state ^13^C NMR spectra of L9, L10, and L11; (**b**) Example of a deconvoluted solid-state ^13^C NMR spectrum (L9).

Solid ^13^C NMR spectra of alfa organosolv lignins L9, L10 and L11 are given in the Figure 1. The spectra present characteristic lignin signals. The pick at 56 ppm is assigned to OMe carbon, lateral chain carbons of phenyl propane units are detected between 60 and 85 ppm, aromatic carbons appear between 100 and 160 ppm. The aromatic region can be divided in three regions as shown in Figure 1: protonated carbons C_AR_–H (105–124 ppm), condensed carbons, C_AR_–C (124–147 ppm) which consist of C1 and carbons involved in cross-linking reactions (β–β, β-5…), oxygenated carbons C_AR_–O (142–155 ppm). Spectra of lignins isolated in presence of Lewis acid are similar but as can be seen from Figure 1, sulfuric acid lignin displays some important differences. In L9, the presence of a sugar contamination is observed at 50–80 ppm (C2–C6) and 105 ppm (C1). Higher number of condensed aromatic carbons (C_AR_–C), associated with higher condensed structures was also observed. In the carbonyl region, a more intense C=O signal at ≈175 ppm was detected for L9. This signal can be assigned to Hibbert’s ketone, arising from dehydration reactions in the lignin lateral chain in acidic conditions.

According to Banoub et al. [12], it could be possible to estimate the proportion of phenylcoumaran units in the lignins from the deconvolution of the ^13^C solid-state spectrum. In wheat straw lignin, the signal at 153 ppm was assigned by the authors to terminal G unit carrying a carboxylic group at C4 and signal at 147 ppm to C3 and C4 of a coniferyl furan-like moiety. The deconvolution of alfa lignin spectra has been performed and an example of a deconvoluted spectrum is given in the Figure 1b. The ratios 147/153 for the three lignins are as following: L9 (147/153 = 2.7) < L11 (147/153 = 4.5) < L10 (147/153 = 5.2). From these data, higher lignin recondensation could be proposed for L10 and L11 with high coniferyl furan-like moiety content. However, important limitations of this method will be discussed in the following section (Section 4).

The sulfuric acid catalyzed organosolv lignins were also analyzed with a ^31^P NMR quantitative method (after phosphitylation) in order to determine the concentration of hydroxyl functional groups. The concentrations of aliphatic and aromatic OH groups of L1 to L9 were 2.5 mmol/g (±1 mmol/g) and 2.1 mmol/g (±0.5 mmol/g), respectively (data not shown). Whatever the severity of the organosolv process, the amount of OH groups in alfa lignin appeared to be comparable to herbaceous organosolv lignin previously described in the literature including wheat straw, switchgrass [24], and miscanthus [8,9].

**Figure 2:**
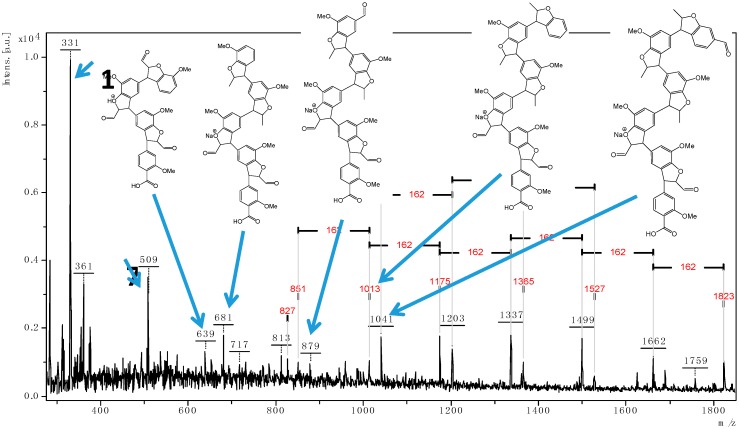
Positive ion MALDI TOF mass spectrum of L9 in the mass range 300–1800 Da.

**Figure 3:**
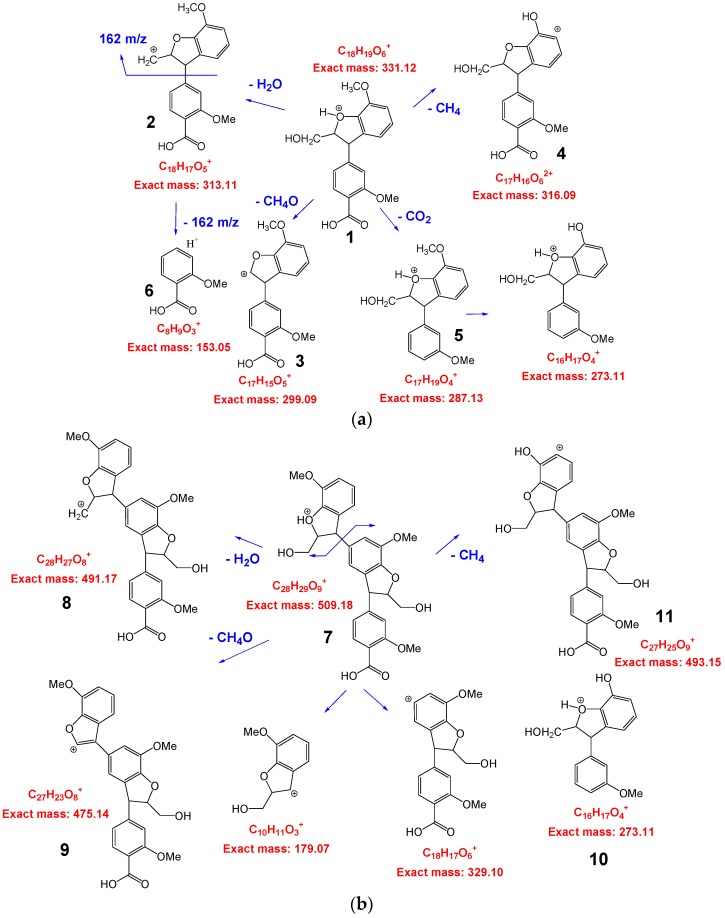
Tentative breakdown processes in the MS/MS of **1** (**a**) and **7** (**b**).

The characterization of series of alfa lignin oligomers were successfully performed by MALDI TOF. The Figure 2 gives the scan range of *m*/*z* 300–1800 Da for L9. As previously observed for wheat straw lignin [12], the most abundant ion over the mass range of *m*/*z* 100–1000 Da was detected at *m*/*z* = 331.11, assigned to a phenylcoumaran derivative **1**. Further structural information regarding this ion was obtained by ASAP-MS/MS analyses. In Figure 3a are tentatively given the molecular structures of the different cations. The precursor ion **1** afforded the product ions **2** (*m*/*z* 313.1), **3** (*m*/*z* 299.1), **4** (*m*/*z* 315.1), **5** (*m*/*z* 287.1) by elimination of water, methanol, methane, and carbon dioxide molecules respectively. The product ion **6** (*m*/*z* 153.1) was produced by a cleavage of the lateral chain generating a phenylcoumaran fragment at *m*/*z* 162. The ASAP-MS/MS analysis of the ion **7** at *m*/*z* 509 was also performed and the formulas are given in Figure 3b. **7** generates the ions **8** (*m*/*z*) 491.1, **9** (*m*/*z*) 475.1, **10** (*m*/*z*) 465.2, and **11** (*m*/*z*) 493.1 by loss of H_2_O, CH_3_OH, CO_2_, and CH_4_ respectively. Elimination of a coumaran moiety from **7** generates also the two ionic fragments **12** (*m*/*z* = 329.1) and **13** (*m*/*z* = 179.1).

From these assignments and based on published data [12], signals at *m*/*z* 639.2, 681.2, 851, 879, 1041, and 1175 were also assigned (Figure 2). All these detected ions displayed five-membered furan-like ring of coniferylic coumaran units, formed by α-5 and β-O-4 linkages with regular and linear structures.

**Figure 4:**
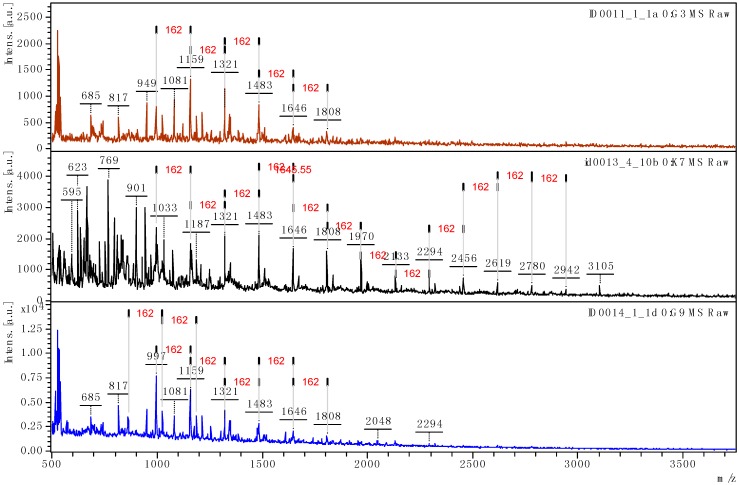
MALDI TOF mass spectrum of L9, L10, and L11 in the mass range 500–3700 Da.

MALDI-TOF spectra of L9, L10, and L11 fractions in the range of *m*/*z* 500–3700 Da are given in the Figure 4. Unlike SEC, MALDI-TOF is not a quantitative technique but it can provide valuable information for unraveling the architecture of lignin molecules. The higher molecular masses were detected for L9 with molecular weight ranging from 400 to 3105 Da. In all the lignins studied, we observed increment peaks of 162 Da relevant of a repetitive de-oxygenated β-5 bonding pattern (Figure 4). This repetitive unit may correspond to a MS/MS fragment of the precursor **1** previously discussed. This pattern is more clearly observed for L9 suggesting a regular structure with high molecular mass fragments (DP ≈ 20). The number of repeating units can be calculated using the expression: DP = [M − 23 (Na^+^) − 2 (end groups)]/162. The repetition of these coumaran units is in accordance with NMR and GPC results and suggests an extensive acid catalyzed repolymerization of the lignin G units during the pulping process. A mechanism of lignin recondensation is proposed in the discussion section. Nevertheless, taking into account the sugar contamination previously observed by NMR and the increment peaks of 162 Da observed, the detection of polyhexose oligomers (glucanes) cannot be totally excluded.

Organosolv lignins L1–L8 were also examined by MALDI TOF in positive and negative mode and similar patterns were observed (MALDI TOF spectra of L2–L6 given in Supplementary Materials, positive mode (Figure S1) and negative mode (Figure S2)). Ions **1** and **7** (*m*/*z* 331 and 509 in positive mode and *m*/*z* 329 and 507 in negative mode, respectively) were detected in all the fractions. ASAP-MS/MS analyses were also performed from these ions (ion **1** from L5 in positive (Figure S3) and negative (Figure S4) mode, ion **7** from L5 in negative (Figure S5) and positive (Figure S6) mode). The collected data attested the presence of polyphenylcoumaran linkages in all the lignin fractions studied. For lignin L5, detected ions at *m*/*z* 1175, 1337, 1499, and 1823 attested the presence of polyhexose oligosaccharides (SP1) as previously reported [25].

**Figure 5:**
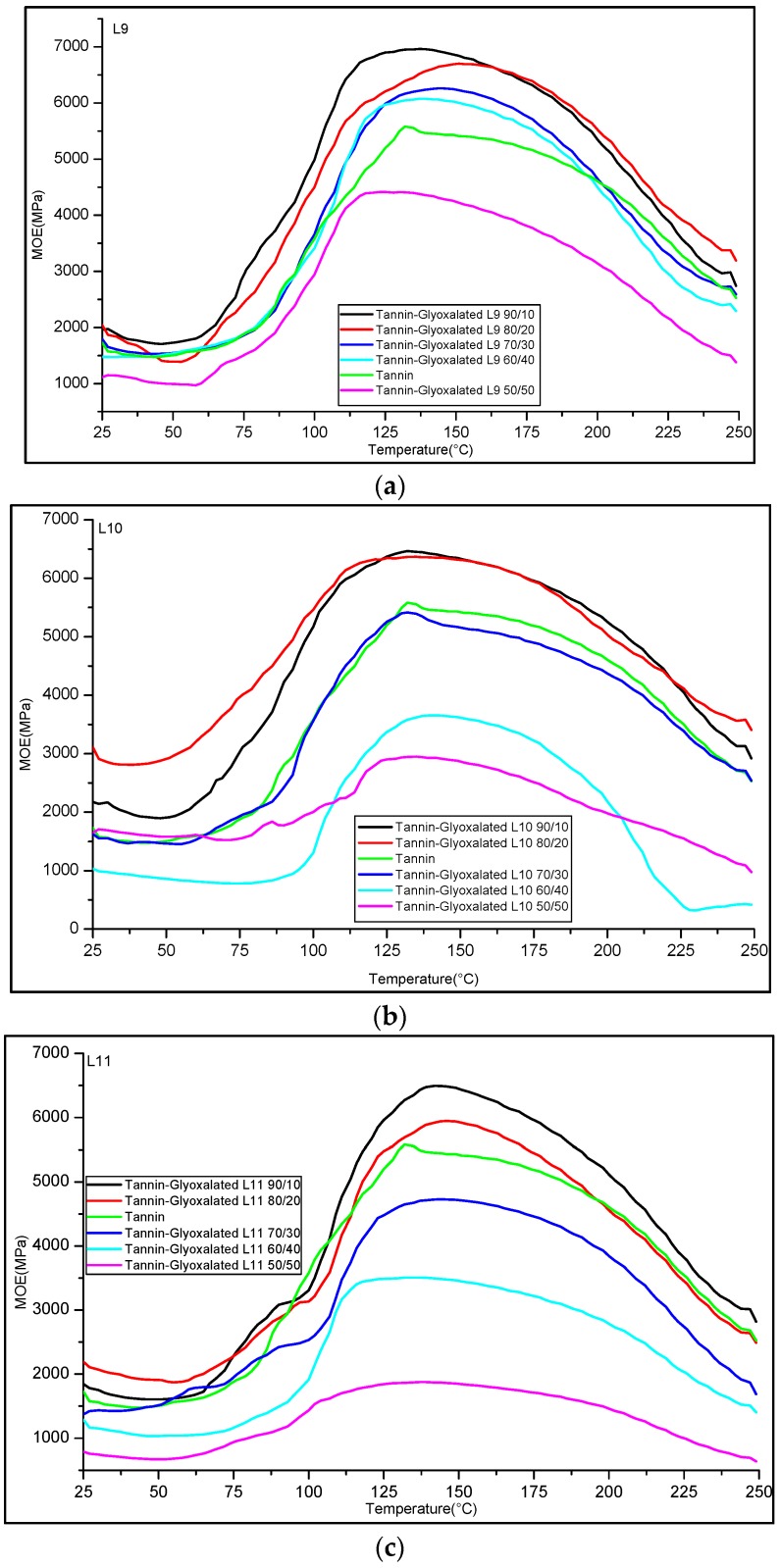
Comparison of thermo-mechanical analysis curing curves of wood joints bonded with lignin/tannins adhesives. Influence of the lignin content; (**a**) L9; (**b**) L10; (**c**) L11.

Thermomechanical analysis is an accurate technique to evaluate the hardening reaction of glue mixes by studying the rigidity of a wood-resin joint as a function of temperature [26]. Adhesive formulations based on a mixture of glyoxalated organosolv lignins/Aleppo pine tannins were prepared and examined by TMA according to a technique already described. The curves of the modulus of elasticity (MOE) as a function of the temperature for L9, L10, and L11 are given in the Figure 5a–c respectively. The influence of the lignin content (from 0% to 50%) in the tannin-based formulation was examined. Surprisingly, compared with pure-tannin resin (MOE_max_ ≈ 5000 MPa), the formulation including 10% to ≈30% of lignin L9, L10, and L11 exhibited better properties with an MOE at 150 °C of = 6000–6500 MPa. However, as previously described, for high lignin contents (40%–50%) a further decrease in the resin properties is observed. This decrease of performance can be rationalized by the lower reactivity of glyoxalated lignin toward cross linking reactions compared to tannins. Interestingly, important differences of reactivity were observed in the TMA study for the three lignin fractions. For L10 and especially for L11, a dramatic decrease of the resins performance was observed with a gradual addition of glyoxalated lignin in the formulation with a MOE_max_ < 3000 MPa for 50% of tannin. On the other hand, L9 adhesive formulation composed of 40% of lignin displayed a good performance with an MOE_max_ nearly 6000 MPa. Thus, from the TMA study, the substitution of ≈40% of tannins by the organosolv glyoxalated lignin L9 seems to have no negative impact on the properties of the resulting resin.

**Figure 6:**
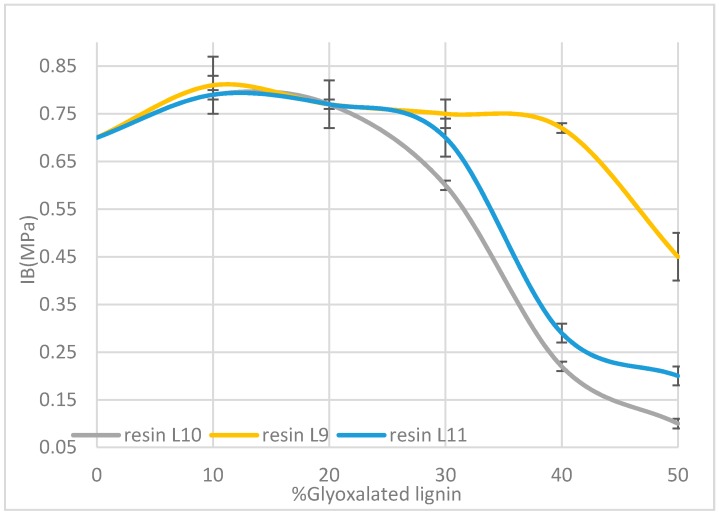
Evolution of the internal bonding (IB) strength of the particleboards as a function of the glyoxatated organosolv lignin content in the formulations.

The tannin/lignin based formulations were tested for application to wood panels. One-layer particleboards were produced at the lab scale and the internal bond strengths (IB) were determined. The IB is a direct measure of the performance of the panel. The Figure 6 gives the evolution of the IB of the particleboards as a function of the lignin fraction (L9–L11) and the lignin content (from 0% to 50%) in the Aleppo pine tannin-based adhesive formulation. The results are in accordance with the TMA study previously exposed and it appears that glyoxalated alfa lignin L9/sumac tannins 50/50 formulation yielded IB which passed relevant international standard specifications for interior-grade panels (IB > 0.35 MPa). In accordance with TMA study, the formulation containing up to 20% of glyoxalated lignin yielded the best performance.

## 4. Discussion

The organosolv lignin can be successfully extracted from alfa grass in presence of acid as catalyst. Good lignin yields (>15% *w*/*w* based on raw material) were obtained with sulfuric acid for combined severity parameters above 3.5. Using these harsh conditions, the recovered organosolv lignin appeared to be highly recondensed. MALDI TOF and NMR experiments showed a high condensed aromatic carbon content. Using a method described by Banoub et al. [12], an estimation of the proportion of phenylcoumaran units in the lignins from the deconvolution of the ^13^C solid-state spectrum was done. It was observed that L9 exhibited the lower condensed structure with a high coniferyl furan-like moiety content. However, this approach and the associated results must be considered with caution. Alfa lignin is an H/G/S type and it has been previously demonstrated by nitrobenzene oxidation that alfa milled ball lignin has a G/S ratio of 1/0.6 [14]. In ^13^C NMR, syringyl units are detected by signals at 154–152 ppm (C3–C5 etherified) and 148 ppm (C3–C5 non-etherified). Thus, etherified and non-etherified syringyl aromatic carbons have some degree of overlap with the signals at 153 and 147 ppm and may distort the data. The lower intensity of the peak at 153 ppm in L9 can in fact be rationalized by an extensive cleavage of S ArOR linkages through acid catalyst hydrolysis of β-O-4 inter-unit linkages.

**Figure 7:**
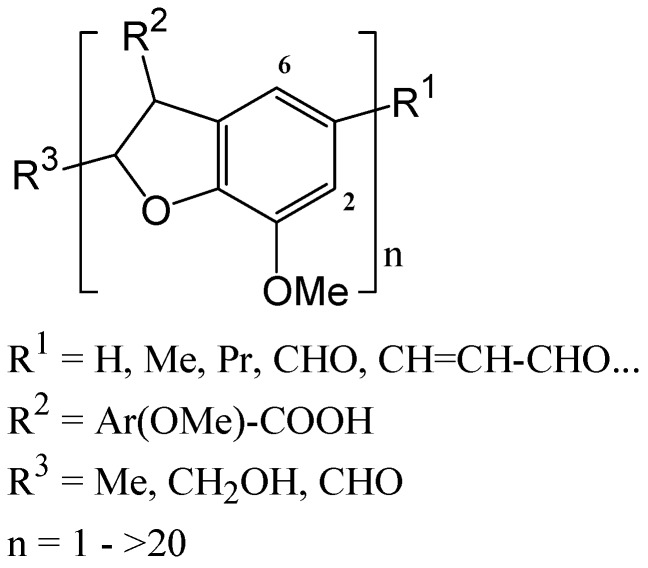
Tentative general formula of recondensed alfa organosolv lignin.

In accordance with the NMR results, the high molecular mass of L9 determined by GPC can be explained by a high repolymerization rate of the lignin through sulfuric acid catalyzed cross linking reactions. The L9 lignin analyzed by MALDI-TOF seems to exhibit a remarkable regular and linear structure given in Figure 7. The MS/MS experiment performed on **1** and **7** allowed an accurate identification of a repetitive de-oxygenated β-5 bonding pattern. The main repeating units in lignin are formed from coniferylic residues linked by a furan-like ring (phenylcoumaran units).

**Figure 8:**
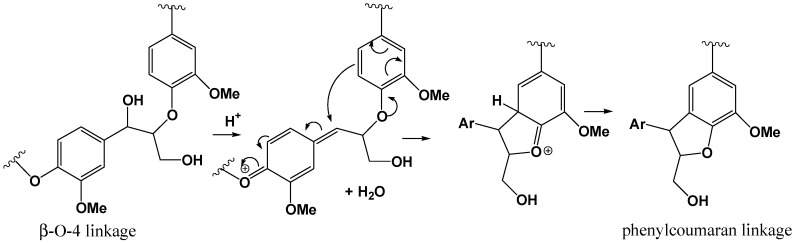
Acid-catalyzed mechanism of formation of a linear polyphenylcoumaran macromolecule.

The regular and linear structure proposed here is not in accordance with the cross-linked 3D network polymer generally described for technical lignin extracted using harsh procedures. However, the organosolv process is described to produce a lignin which is less modified than other pretreatment lignins, relatively close to native lignin which is a linear oligomer rather than a network polymer [27]. Such a linear structure has already been described for wheat straw organosolv lignin [12,13]. A tentative acid-catalyzed mechanism of formation of a linear polyphenylcoumaran macromolecule from β-O-4 linkages is given in Figure 8. The utilization of Lewis acid as catalyst for the organosolv extraction induces important modification regarding the recovered lignins structure compared to sulfuric acid. As previously described, the cleavage of β-O-4 linkages was the major mechanism of lignin breakdown and is a function of the hardness of the catalyst [8,9]. Lower recondensation rates were also observed with the Lewis acids. Thus, lignins exhibiting lower molecular mass and lower condensed carbons C_AR_–C (e.g., β-5 coumarane moiety, Figure 7) were isolated.

Nevertheless, it must be specified that the linear lignin described here by MALDI TOF analysis corresponded to lignin fragments produced by the laser desorption of the raw lignin sample. It should be noted that the presence of aromatic OH groups detected by ^31^P NMR (2.1 mmol/g) also suggest the presence of open lignin structures not detected by this technique. 

It is well known that lignin is of a much lower reactivity than flavonoid tannins toward cross linking reactions. In this study, organosolv lignin was pre-activated with glyoxal prior to the preparation of the formulations in order to increase its reactivity toward tannins through the introduction of methylol-type groups on the aromatic moieties [28]. However, the reactivity of the activated lignin is still too slow to cross-link alone in the conditions of the experiments (short press times). This can explain the decrease of the resins MOE and of the panels IB with a relative high proportion of tannins in the formulations (40%–50%). Surprisingly, addition of a small amount of organosolv alfa lignin (up to 20% for L10 and L11, 30% for L9) in a tannin-based adhesive seems to improve its performance. This was observed for the three lignin fractions studied (L9, L10, and L11) in the TMA experiments performed from the resins and in the performance of the corresponding produced panels (IB).

In this study, for the first time we showed the impact of the lignin extraction conditions on the performance of the lignin-based resins produced for adhesive applications. Compared to L10 and L11, lignin L9 extracted in presence of sulfuric acid yielded adhesives with higher performance using high lignin content (50%). As previously demonstrated, L9 was isolated at higher severity and is highly recondensed with the presence of regular structures composed of furan-like rings. The partly linear and polycyclic structure of L9 lignin could justify a higher reactivity toward electrophilic substitution with glyoxal because of the relatively low steric hindrance of the aromatic ring (Figure 7). The involvement in the cross linking reactions of phenolic open lignin structures detected by ^31^P NMR can also be proposed according to well-known mechanisms [1,2].

## 5. Conclusions

The extraction of alfa ethanol organosolv lignin was performed and the influence of the organosolv process conditions (pulping severity and nature of the acid catalyst) on the lignin structure was investigated. The organosolv alfa lignin was characterized for the first time by MALDI-TOF MS/MS, ^13^C NMR, and SEC. It was demonstrated that: (1) the lignin isolated when using sulfuric acid as catalyst exhibited a highly recondensed structure and contained linear macromolecules formed by polyphenylcoumaran linkages (DP ≈ 20); (2) the addition of 10% to 30% of organosolv alfa lignin in a tannin-based formulation improved the adhesive performance ; (3) the catalyst used for the lignin extraction impacted the resin performance, quignard; (4) a formulation composed of 50% glyoxalated alfa lignin and 50% of Aleppo pine tannins yielded good internal bond strength results for the particleboard and satisfied relevant international standard specifications for interior-grade panels.

## Figures and Tables

**Table 1:** Experimental conditions of the organosolv pretreatment evaluated for alfa, lignin yields, and molecular mass.

Experiment	Catalyst	Conditions	CS ^a^	Yield % ^b^	*M*_w_^c^
L1	H_2_SO_4_ 0.7%	160 °C, 1 h	1.47	1	11,235
L2	H_2_SO_4_ 0.2%	167 °C, 1 h	1.15	2.8	12,382
L3	H_2_SO_4_ 0%	185 °C, 1 h	1.08	7.6	6541
L4	H_2_SO_4_ 0.7%	185 °C, 1 h	2.20	16.22	7059
L5	H_2_SO_4_ 1.4%	185 °C, 1 h	2.80	18.66	8630
L6	H_2_SO_4_ 0.2%	203 °C, 1 h	2.22	16.33	4469
L7	H_2_SO_4_ 1.2%	203 °C, 1 h	3.12	15.08	4625
L8	H_2_SO_4_ 0.7%	210 °C, 1 h	2.94	10.12	6873
L9	H_2_SO_4_ 0.12%	160 °C, 2 h, pH 2.4	1.45	13.22	12,330
L10	Sc(OTf)_3_	160 °C, 2 h, pH 2.4	1.45	10.43	4360
L11	FeCl_3_	160 °C, 2 h, pH 2.4	1.45	10.11	8306

^a^ Combined severity = log [*t* × exp((*T* − 100)/14.7)] − pH; ^b^ lignin yield based on dry raw material; ^c^ molecular weight determined by GPC analysis.

## Supplementary Materials

Supplementary Materials are available online at www.mdpi.com/2073-4360/8/9/340/s1.
