# Supplementary material for: Organosolv Lignin-Based Wood Adhesive. Influence of the Lignin Extraction Conditions on the Adhesive Performance
Source: Polymers (Basel). 2016 Sep 14;8(9):340. doi: 10.3390/polym8090340 (PMC6431968; doi:10.3390/polym8090340)
Supplement: Supplementary file 1 [file polymers-08-00340-s001.pdf]

Supplementary Materials: Organosolv Lignin-Based Wood Adhesive. Influence of the Lignin Extraction Conditions on the Adhesive Performance

Issam Dababi, Olinda Gimello, Elimame Elaloui, Françoise Quignard and Nicolas Brosse

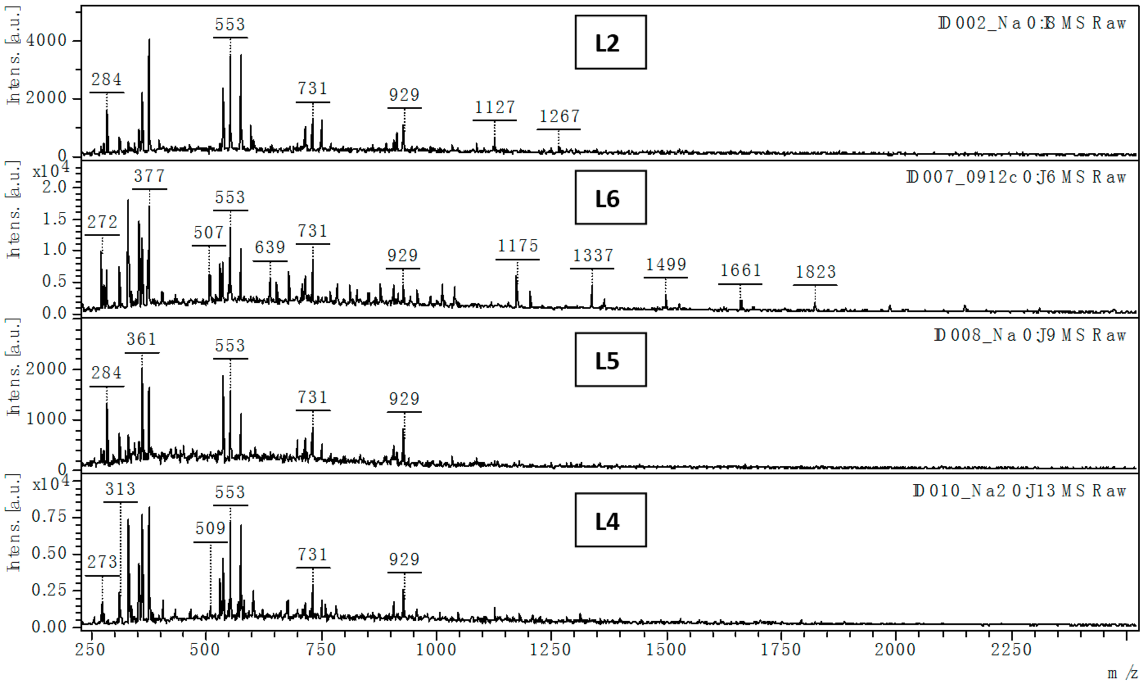

Figure S1. Positive mode MALDI TOF spectra of lignins L2, L4, L5, L6.

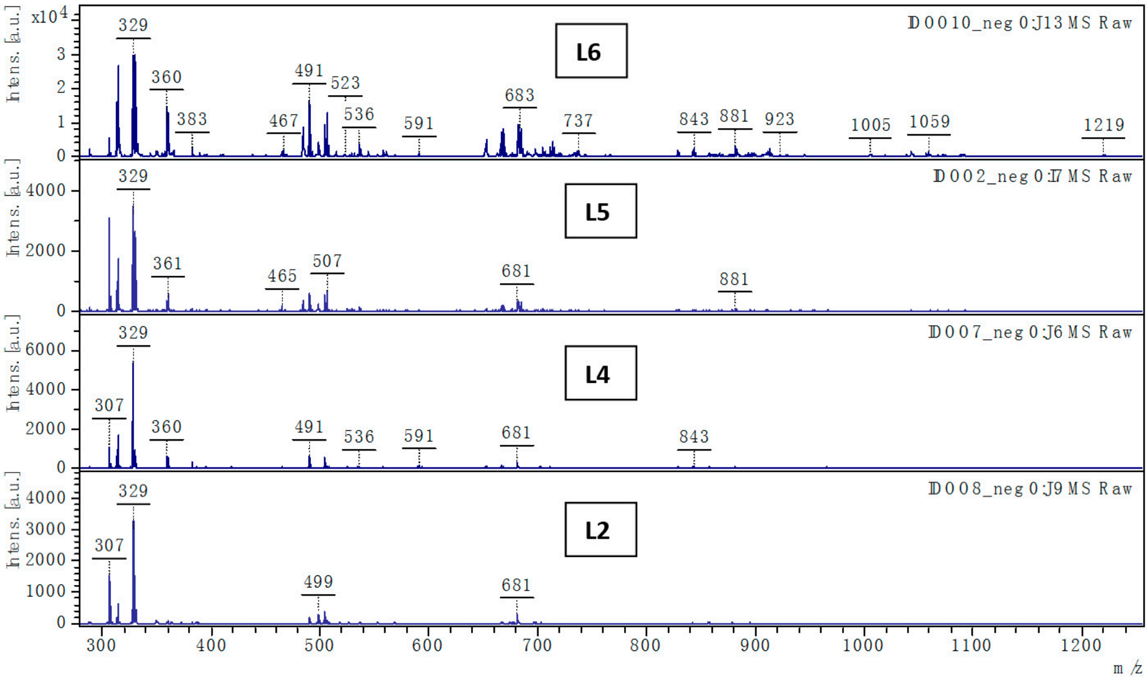

Figure S2. Negative mode MALDI TOF spectra of lignins L2, L4, L5, L6.

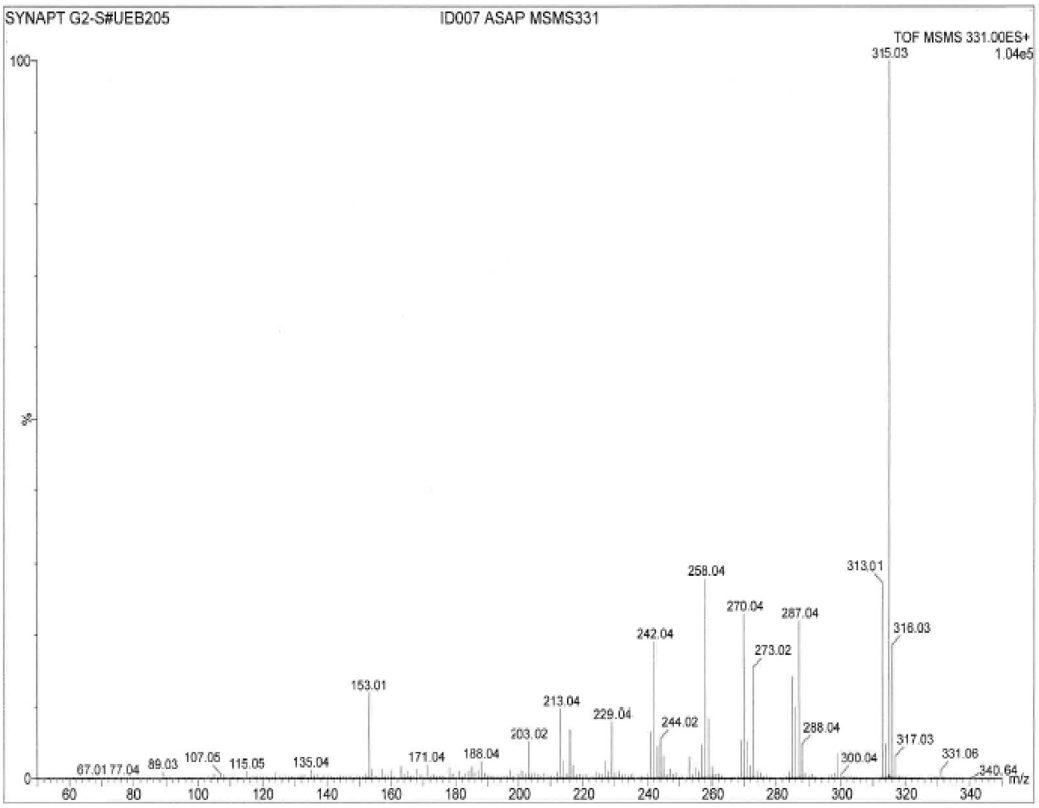

Figure S3. ASAP-MS/MS spectrum in positive mode of ion **1** from L5 (*m*/*z* 331).

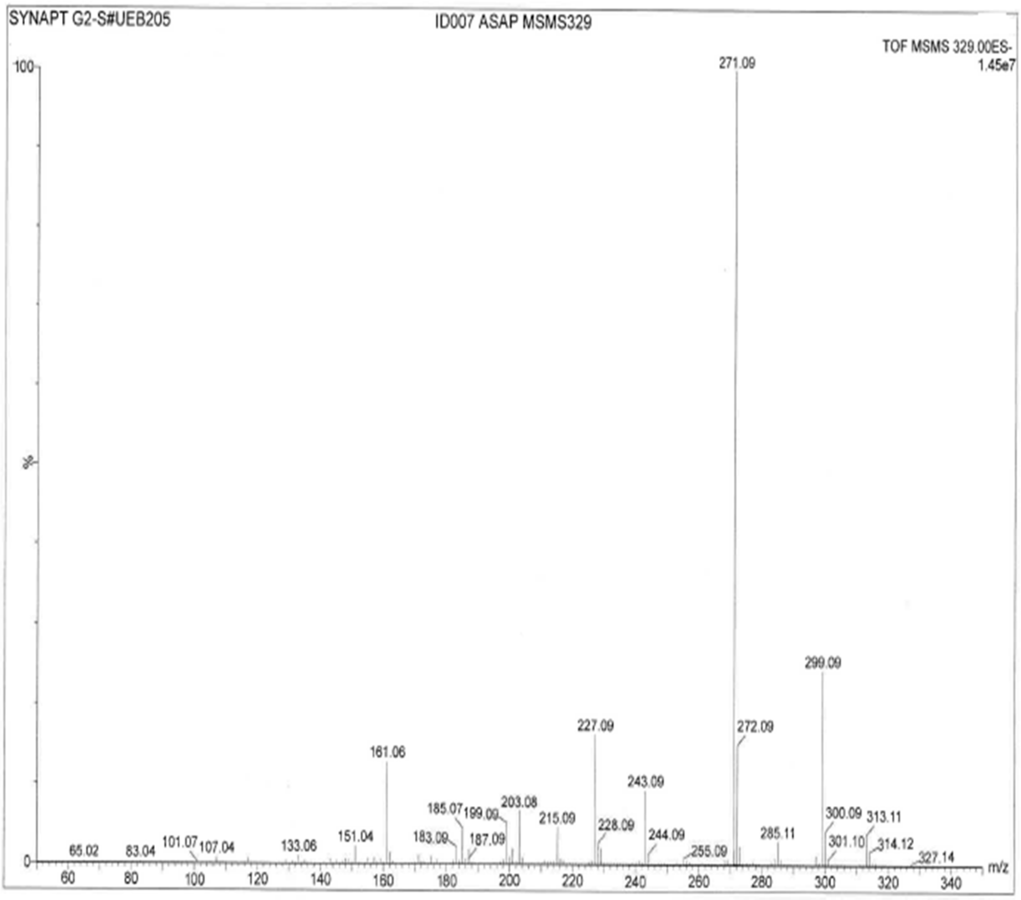

Figure S4. ASAP-MS/MS spectrum in negative mode of ion **1** from L5 (*m*/*z* 329).

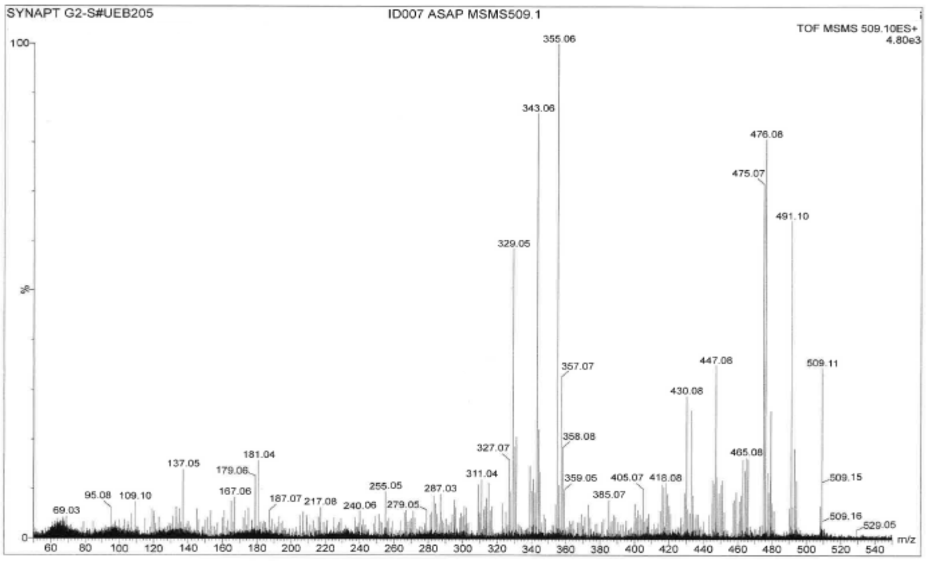

Figure S5. ASAP-MS/MS spectrum in positive mode of ion **7** from L5 (*m*/*z* 509).

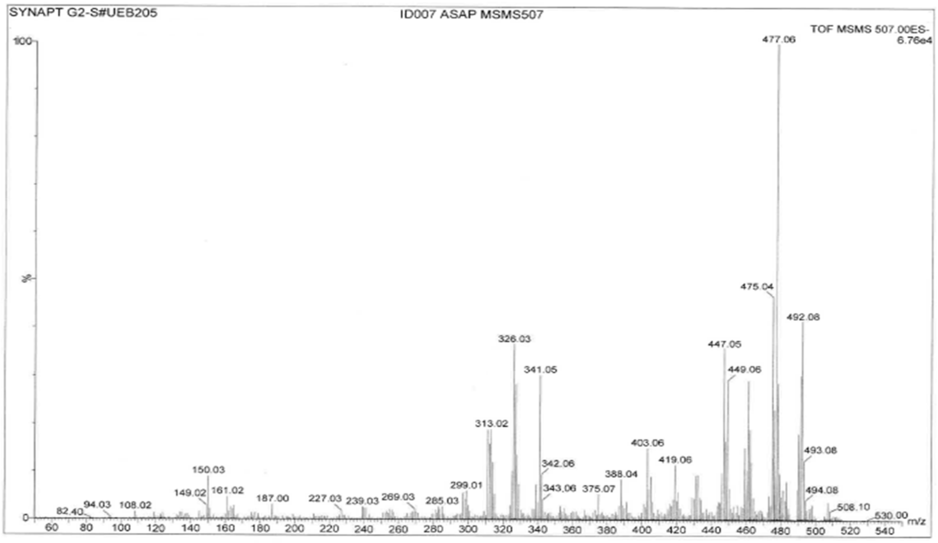

Figure S6. ASAP-MS/MS spectrum in negative mode of ion **7** from L5 (*m*/*z* 507).
